# Optimisation of the Green Process of Industrial Hemp—Preparation and Its Extract Characterisation

**DOI:** 10.3390/plants11131749

**Published:** 2022-06-30

**Authors:** Taja Žitek, Petra Kotnik, Teo Makoter, Vesna Postružnik, Željko Knez, Maša Knez Marevci

**Affiliations:** 1 Faculty of Chemistry and Chemical Engineering, University of Maribor, Smetanova ul. 17, SI-2000 Maribor, Slovenia; taja.zitek@um.si (T.Ž.); petra.kotnik@um.si (P.K.); vesna.postruznik@um.si (V.P.); zeljko.knez@um.si (Ž.K.); 2 Faculty of Medicine, University of Maribor, Taborska 8, SI-2000 Maribor, Slovenia; 3 Faculty of Mechanical Engineering, University of Maribor, Smetanova ulica 17, SI-2000 Maribor, Slovenia; teo.makoter@student.um.si

**Keywords:** supercritical fluid extraction, ultrasonic extraction, melanoma cells, WM-266-4

## Abstract

Natural medicines and products are becoming increasingly important in the pharmaceutical and food industries. The most important step in obtaining a natural remedy is the processing of the natural material. This study offers the separation of the industrial hemp plant into fractions by mechanical treatment, which has a significant impact on the selectivity of the obtained fractions. This study also offers a solution to reduce waste by fractionating industrial hemp, focusing on the fraction with the highest cannabinoid content (49.5% of CBD). The study confirmed the anticancer potential of the extract, which prevents further division of WM-266-4 melanoma cells at a concentration of 10^−3^ mg/mL. However, application of the extract (c = 10^−3^ mg/mL) to normal human epidermal melanocytes proved to be insignificant, as the metabolic activity of the cells was the same as in the control cell group.

## 1. Introduction

Knowing the phytotomy of the hemp plant and understanding its potential in phytopharmacy can help us to select the proper part of a certain species and obtain a high-quality extract or product. Hemp has great added value as each part of the plant represents many potentially valuable resources for quality products. The hemp plant consists of the woody part (44% of the plant weight), fibres (24%), seeds (11%) and other components such as flowers, leaves and dust (21%) [1,2,3]. This diversity of substances means that the potential uses of hemp go far beyond medicinal use, since every part of the hemp plant can be useful. A large part of the plant is made up of the fibres extracted from the stems, which, with low weight and high strength, are an important material for the construction industry and offer good prospects for the automotive industry [4,5]. The seeds can also be used in a variety of applications, such as for the production of cooking oil, which have been shown to have several beneficial effects on the body [6,7,8]. Hemp seeds are also used to make biodiesel through seed pressing. They have been shown to contribute four times more fuel than soybeans, which has led the United States to produce biofuels [9,10].

Hemp has great potential for the sustainable planning of whole plant processing according to the zero waste and green process concept. Since each part of the hemp has its own potential for a certain product, the main thing in planning is the pretreatment of the material (for example, the screening method), which separates plant parts. This pretreatment process allows fragmentation of materials according to their active size and consequently, after extraction, the desired components [11]. The entire concept results in extracts with a high proportion of the desired components and above all, sustainability. One of the most important variables that affects the quality of the product, besides the appropriate choice of a plant screening method, is the selection of an extraction method. The choice of extraction solvent is crucial. Studies have already introduced several solvents such as methanol, ethanol, chloroform, butane, hexane, etc. [6,11,12]. However, there are safety reasons regarding their toxicity, thus it is important to comply with the European directive (Directive 2009/32/EC of the European Parliament and of the council of 23 April 2009 on the approximation of the laws of the Member States on extraction solvents used in the production of foodstuffs and food ingredients), which recognises the extraction solvents used in the production of food and food ingredients (such as ethanol and carbon dioxide) [13].

In this study, the investigation into the pretreatment of the material was focused further on the selected segments obtained after processing, which were obtained mainly from hemp flowers and leaves. The female cannabis plants were used as male plants do not produce flowers, and are the main topic of this work. Certain known forms of leaves also constitute a part of the plant and are located above the cola from which the flower emerges [4]. Within the cola area, tiny orange, brown hairs are positioned that sprout from the flower and the small nodules (calyx) from which the flower emerges. The nodules usually have an extensive collection of trichomes, which are glands that secrete cannabinoids [4,14]. Shiny crystals on tiny leaves (also called sugar leaves) are crystallised secretions of terpenes, tetrahydrocannabinol (Δ9 THC), cannabidiol (CBD) and other cannabinoids [15,16,17]. Phytocannabinoids represent a group of C21 or C22 terpenophenolic compounds synthesised from fatty acid precursors [18] in the acid (cannabinoid) form (C22). They are decarboxylated to their neutral forms (C21) upon exposure to light [19]. Cannabigerolic acid (CBGA) is a major precursor of tetrahydrocannabinolic acid (Δ9-THCA), cannabidiolic acid (CBDA) and cannabichromenic acid (CBCA). Geranyl diphosphate and olivetolic acid are synthesised to CBGA by synthase [20]. CBGA, CBDA and CBCA are formed by various cyclisations and have pentyl side chains (C5-phytocannabinoids). Decarboxylation of these precursors results in Δ9-THC, CBD, CBC and its chemical artifact CBL (cannabicyclol). CBN (cannabinol) is formed by the degradation of THC. On the other hand, cannabigerovaric acid (CBGVA), Δ9-tetrahydrocannabivaric acid (Δ9-THCVA), cannabidivaric acid (CBDVA), and cannabichromevarinic acid (CBCVA) are formed from geranyl diphosphate and divaric acid [20]. Nonacidic forms of cannabinoids (CBD, THC, CBG, etc.) are credited with many healing effects on the human body [1,21]. The extraction of raw material results in the acidic form of cannabinoid compounds and decarboxylation is required to obtain nonacidic forms [22].

As mentioned above, in the first step we focused on the research of the pretreatment material (screening) for the separation of plant parts. There has been no such precise separation of dried material described in the literature. The separation (sieving) was used to achieve higher selectivity of the required components (cannabinoids) already in the crude mass. The crude mass was further extracted in two unconventional ways with different solvent polarities (ethanolic ultrasonic extraction and supercritical CO_2_ extraction) and the resulting extracts were mixed in a 1:1 ratio. The selected extraction procedure and mixing were explained as the most appropriate in the previous study [23]. The CO_2_ solvent was chosen because it isolates nonpolar components, such as cannabinoids in case of hemp extraction. On the other hand, ethanol as a polar solvent isolates other components, as reported by Appendino et al. who studied the isolation of the polar cannabinoid carmagerol [24]. They point out that previous research focused on a specific polarity range, which may have overlooked smaller compounds with higher or lower polarity than the major cannabinoids. However, no co-solvent was added to the supercritical CO_2_, otherwise it could increase its solubility in favour of other polar molecules that are not desired; higher solvent strength could mean lower process selectivity [23,25]. The aim of this study was to demonstrate that appropriate pretreatment affects the better selectivity (purity) of the extract and improves anticancer activity while bioavailability is increased. Therefore, the research was conducted on melanoma cells WM-266-4 and normal human epidermal melanocytes, demonstrating the biocompatibility of the extract.

## 2. Materials and Methods

Hemp (*Cannabis sativa*) was purchased from a local grower in Slovenia (Makoter agricultural estate, Cven, Slovenia, coordinates: 46.5431403, 16.2197896). The used hemp type was *Kc Dora* with an organic certificate (BV-SVN-EKO-160/20). The material was supplied dried. The screened parts of hemp used in this study were also prepared on the growers’ estates.

### 2.1. Pretreatment of Hemp

**Figure 1:**
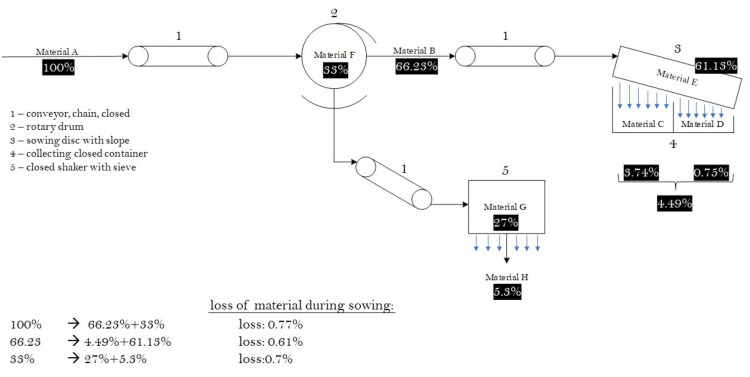
The process of sieving and separating parts of hemp. Material A: whole dried hemp; B: dried seedless and wasteless hemp; C: hemp powder - the first half of the sifted material in a sieve (3); D: hemp powder - the other half of the sifted material in a sieve (3); E: material residue on sieve (3); F: material remaining in the sieve (2); G: pure hemp seeds and H: waste material (mainly stems).

The dried hemp plant (stems, leaves and flowers) was sieved according to the procedure presented in Figure 1. It represents the output of each sieving unit and the loss of the material during the procedure. Material A (on Figure 1) represents the entire dried plant (stems, leaves and flowers), which goes along the closed conveyor belt (1) to the rotating drum (2). In (2), the stems and seeds in the drum (material F) are separated from the other parts of the hemp. The rest of the material (material B) goes along the conveyor belt (1) to the sieve with a slope (3), under which the collecting vessel (4) is separated into two parts. In the first part, material C is collected, and in the second, material D. The vessel is separated, because more cannabinoids are expected to fall in the first half than in the second. The material remaining on the sieve (3) represents material E. The material F that remains in the rotating drum is transferred along the conveyor belt (1) to the shaker with a sieve (5), where the waste material (material H) is separated from the seeds (material G).

Seven material samples (A, B, C, D, E, G, H) were obtained during sieving. Dried hemp (materials A, B, C, D, E, and H) was decarboxylated for 60 min at 140 °C. Extraction was performed for materials A, B, C, D, E, and H. Material F was a collection of seeds and waste material, which was further sieved (5) and only then the resulting hemp seeds (material G) and waste material representing stems (material H) were extracted. Hemp seeds do not contain cannabinoids, but the extraction of this fraction has been performed for comparison. 

### 2.2. Extractions

**Figure 2:**
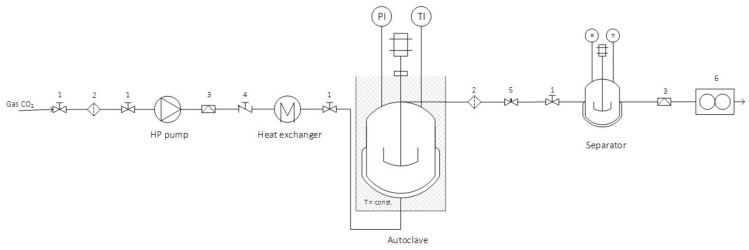
Supercritical fluid extraction system; 1: valve, 2: high pressure filter, 3: rapture disc, 4: one-way valve, 5: regulating valve, 6: gas flowmeter, PI: pressure indicator and TI: temperature indicator.

The subsequent extraction procedure was explained and the conditions for the extraction were already established in our previously published article [23]. Supercritical fluid extraction with CO_2_ and ultrasonic extraction with EtOH were carried out according to the procedure described by Žitek et al. [23]. However, once both extracts were obtained, they were mixed in a 1:1 ratio. The ultrasonic extraction process was performed at 40 kHz at 25 °C. Solvent (EtOH) was removed at 40 °C under reduced pressure with a rotary evaporator (Büchi Rotavapor R-114, Flawil, Switzerland). On the other hand, the supercritical experiments were performed in an SFE system, shown in Figure 2. Material (10 g) was placed in an autoclave, and extraction was carried out at 350 bar and 60 °C. The solvent to feed ratio was 8.205. Extraction procedures were performed in triplicates. The obtained extracts were stored at −20 °C until analysed with LC-MS/MS. 

### 2.3. Determination of Cannabinoids with LC-MS/MS Method

An Agilent 1200 HPLC apparatus coupled with an Agilent 6460 Jet Stream triple quadrupole (QQQ) mass spectrometer was used in this study. Using a chromatographic Agilent Poroshell EC-C18 column with 2.7 μm particles and dimensions of 100 × 2.1 mm ID after an Agilent Poroshell EC-C18 precolumn with 4.6 μm particles, separation of cannabinoids was achieved with a mobile phase of water containing 0.1% formic acid (A) and acetonitrile containing 0.1% formic acid (B). The initial conditions were 34% of B held for 8 min; then, B was increased to 95% over 4 min and maintained for 1 min; then, B was reduced to 34% over 1 min and maintained for 6 min with an additional 3 min post-run. The flow rate was 0.2 mL/min, and the column temperature was maintained at 35 °C. Detection was performed in negative ion mode, and analytes were ionised by electrospray and monitored in multiple reaction monitoring (MRM) mode. Optimised mass spectrometer parameters were: gas temperature 300 °C, gas flow 5 L/min, nebuliser voltage 35 V, sheath gas temperature 250 °C at flow 11 L/min, and capillary and nozzle voltage 4000 V and 500 V, respectively. The MRM transition ions are shown in Table 1.

### 2.4. Metabolic Activity of Metastatic Melanoma Cells WM-266-4 and Normal Human Epidermal Melanocytes upon Application of the Extracts

Different concentrations of the extracts in DMSO (c = 60, 30, 20, 10, 5, 1, 0.5 and 0.1 mg/mL) were applied to melanoma cells WM-266-4. After the initial results, the experiments were repeated in a lower concentration range (with small differences in the concentrations) and the extract was applied to healthy cells, melanocytes.

The skin metastatic melanoma cell line WM-266-4 (ATCC^®^ CRL1676™, Manassas, VA, USA), purchased from the American Type Culture Collection, was grown in a complete medium containing Eagle’s Minimum Essential Medium (EMEM, ATCC^®^ 30-2003™, Manassas, VA, USA) with 10% fetal bovine serum (FBS, ATCC^®^ 30-2021™, Manassas, VA, USA) and 0.02% MycoZap™ Plus-CL (Lonza, Portsmouth, NH, USA) and incubated at 37 °C, 5% CO_2_, ≥90% RH. The cells were plated at a density of 1 × 10^4^ viable cells per well in 96-well culture plates and cultured for 24 h to allow cell attachment.

**Figure 3:**
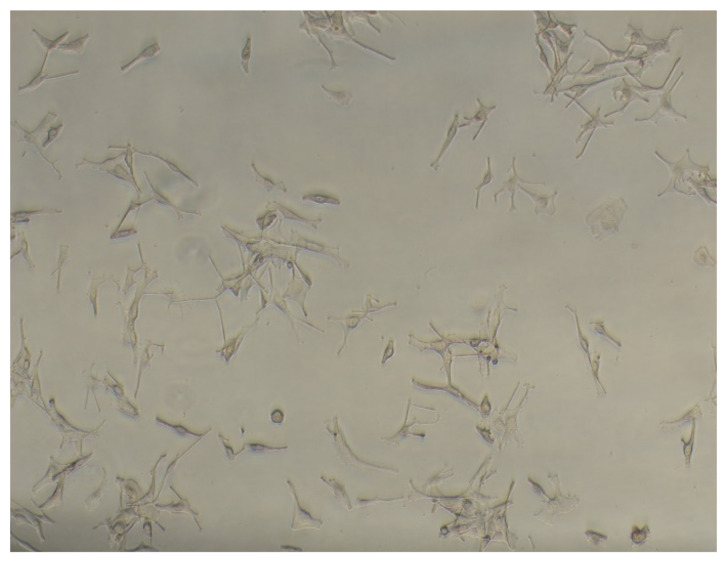
Normal human epidermal melanocytes (NHEMs) during cultivation.

Normal human epidermal melanocytes (NHEMs) (SI-104-05A, Taufkirchen, Germany) are primary cells. The cells were grown in a complete medium: melanocyte growth medium (SI-135-500, Manassas, VA, USA). The cells were plated at a density of 1 × 10^4^ viable cells per well in 96-well culture plates and cultured for 24 h in medium to allow cell attachment (Figure 3).

Five replicates of each experiment were performed. To measure the metabolic activity of the cells, they were exposed to selected concentrations of extracts and cultured for 24 h. Control cells were cultured for the same time and under the same conditions, but without the addition of extracts. A WST-8 Colorimetric Cell Viability Kit I (PromoKine, PromoCell, Heidelberg, Germany) was used according to the manufacturer’s instructions. Absorbance was measured spectrophotometrically at 570 nm (background absorbance at 630 nm) for all samples in pentaplicate. The percentage of metabolic activity of the cells (MA) was calculated according to a procedure described in a previous study [23]. After application of extracts, the cell morphology was observed using an inverted microscope (DM16000B, Leica, Morrisville, NC, USA) with a digital camera (DFC365 FX Leica, Buffalo Grove IL, Leica, Morrisville, NC, USA).

### 2.5. Detection of Cell Apoptosis

To determine the level of advancement of the apoptosis process, a Muse Cell Analyzer and Muse Annexin V & Dead Cell Kit (Luminex, Commercial Ave, Northbrook, IL) were used. Analysis was performed using a dead cell marker and calcium-dependent phospholipid-binding protein Annexin V and 7-AAD according to the manufacturer’s instructions (Muse Annexin V & Dead Cell Kit Catalog No. MCH100105). Briefly, after each experiment, cells were trypsinised and 100 μL of cells suspensions were prepared for analysis. Next, 100 μL of Annexin V & Dead Cell Reagent was added to each sample and mixed. Samples were stained for 20 min in the dark and then analysed with the Muse Cell Analyzer. Each experiment was performed in triplicate and the mean value was determined.

### 2.6. Statistical Analysis

Statistical analysis was performed using R software version 4.1.0. and RStudio Version 1.4.1717 supported by the following packages: rstatix [26], ggplot2 [27] and dplyr [28]. Differences in melanoma cell metabolic activity between extract groups were evaluated, as well as the correlation between extract concentration and cancer cell metabolic activity. The Shapiro–Wilk test for normality of distribution was performed (*p* = 0.010). As the data were not normally distributed, the Kruskall–Wallis test was selected to evaluate the differences in metabolic activity between extract groups. The Spearman correlation test was performed to evaluate correlation between extract concentration and cancer cell metabolic activity. Numerical variables with abnormal distribution are described by median (interquartile range) [29].

## 3. Results and Discussion

This study is oriented towards a sustainable concept of utilisation of the whole hemp plant by integrating a pretreatment process to separate different parts of the hemp plant. This results in the high content of cannabinoids in specific fractions such as fractions B, C and D. This is a prerequisite for a high-quality extract with specific components.

Table 2 and Figure 1 show percentages based on the input material (material A), representing 100% of the material. Figure 1 shows that material A, constituting 100% of the weight, was placed in a rotary drum (2). In total, 66.23% of the material was sieved through the sieve (2), and 33% remained in the drum. In the first stage of sowing, 0.77% of the material was lost. The mass that fell through the first sieve (2) was sieved through a second sieve, a sowing disc with slope (3), leaving 61.13% on the sieve (3). In addition, 4.49% of the material fell separately in two parts into the collection container (4). The first part of the collection container (4) contained 3.74% of the material, and the second part contained 0.75% of the material. The loss of material in this stage was 0.61%. In the third screening stage, the material F that remained on the drum when screened through the sieve (2), i.e., 33%, was screened through a closed shaker with a sieve (5). Out of this, 5.3% was sieved and 27% remained on the sieve (seed). In the last stage, 0.7% of the material was lost.

It was observed that the loss of material during seeding was relatively low (2.08%). Therefore, by analysing the material loss, it was proved that the process itself is economical in terms of material loss.

Table 3 shows yields after extraction. Our assumptions about the maximum content of cannabinoids in the extract, obtained from material C, were confirmed by the LC-MS/MS analysis (Table 4). Despite the process of separation of hemp parts resulting in the lowest amount of fraction C (Table 3; 3.74%), the yield was significantly higher (Table 3); 19%. It was also found that the extracts obtained from material C contained the highest levels of cannabinoids, especially CBD components (EC = 49.5%).

Table 4 presents the cannabinoid contents in hemp extracts (percentage of selected cannabinoids (CBD, THC) per gram of extract. The ratios of cannabinoids in the plant and later in the extract depend on the type of plant, harvest, weathering, etc. [30]. Therefore, the results are difficult to compare with other studies because the literature is scarce on studies of the separation of a plant into fractions. Nevertheless, in general, our results can be compared with the literature based on material A and material E. It is reported that the content of CBD in the extracts after decarboxylation is between 30% and 40% [31,32]. In this study, the content of CBD in the hemp extract from material A was EA = 27.1%, whereas the content of CBD in extract from material E was 36.8% (EE). The higher contents were achieved in extracts from materials B, C and D, where almost 50% of the CBD component was measured in the hemp extract from material C (EC).

The increasing need for recycling and supplies from the planet is met by this novel approach, the screening process, which represents the innovativeness of this process. It is essential that a minimum amount of solvent is used in the recovery process and to have as little waste as possible. Therefore, as a solution, we present the process of sieving hemp, where every fraction can be used.

According to the results, the most suitable materials for extraction are material C and material D (for extraction of world-famous hemp resin). Material E would be suitable for use as tea, as it retains enough cannabinoids despite sieving [33]. Hemp oil, which has been known on the market for some time, is obtained from material G [34]. The waste, material H, would be interesting to research further in terms of fibre content. Hemp fibres are used in technical textiles (ropes, nets, tents, sails, carpets, etc.), textiles (clothing, footwear, tablecloths, bedspreads, bedding, etc.), industry and construction (geotextiles, bio composites, nonwovens, pipes, moulding, insulation, etc.) [9,10,35]. Stems can also be used for energy and in the environment (biofuel, ethanol, anti-erosion textiles), paper industry (cardboard, fine/coarse paper, filters), agriculture (mulch, animal litter), etc. [36,37]. In this study, the focus was on the fraction that contained the most cannabinoids (EC). In the future, it would be interesting to study the benefits of other fractions for humans.

### Effect of the Extract on Metastatic Skin Cancer Cells and Normal Human Epidermal Melanocytes

**Figure 4:**
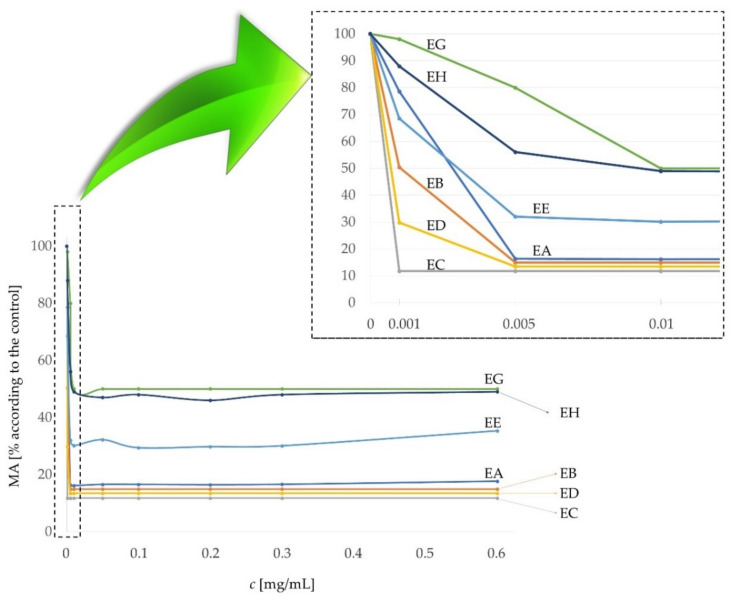
Metabolic activity of melanoma cells WM-266-4 at different concentrations of extracts (EA, EB, EC, ED, EE, EG and EH).

The extracts (EA, EB, EC, ED, EE, EG and EH) were applied to WM-266-4 melanoma cells at different concentrations. The minimum concentration at which significant inhibition of cancer cell division or activity occurred was 0.005 mg/mL. The exception was the EC extract, where the required concentration was even lower than in the other cases (0.001 mg/mL). At a concentration of 0.001 mg/mL, the metabolic activity of cancer cells was only 11.74% compared to the control (Figure 4). ED and EB extracts also showed a significant decrease in cancer cell function at a concentration of 0.001 mg/mL (Figure 4), but with slightly higher percentages of cell metabolic activity (approx. MA (ED) = 30% and MA (EB) = 50%) than at higher concentrations (e.g., 0.005 mg/mL).

**Figure A1:**
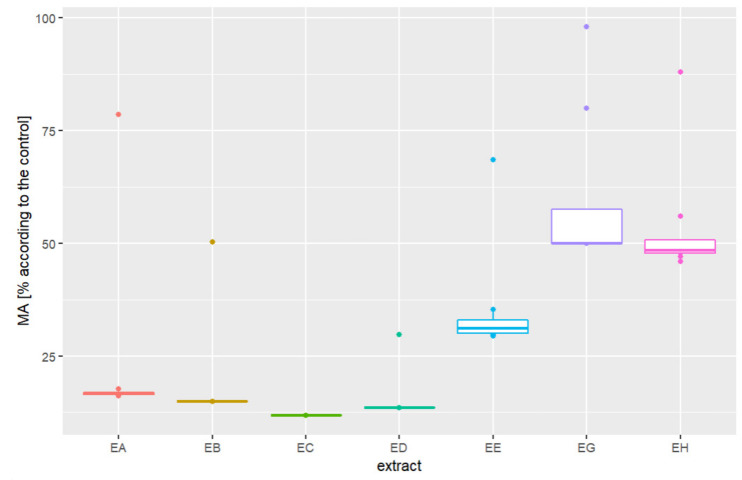
Comparison of cancer cell metabolic activity in % according to control between different hemp extracts.

The Kruskall–Wallis test confirmed significant differences between the metabolic activity of cancer cells after application of different extracts (H(6) = 45.264, *p* < 0.001). Regardless of the added concentration of the extract, the metabolic activities of the cancer cells differed from each other with respect to the added extract, as shown in Figure 4 and Figure A1. The median MAs of the samples EA, EB, EC and ED were below 20% of MA according to control. Cell growth was most inhibited by the EC extract, which had a median of 11.7 (11.7, 11.7)% of MA according to control. The EE extract had a median of 31.1 (30.0, 33.0)% of MA according to control. Least effective were the EH extract with a median of 48.5 (47.8, 50.8)% of MA and EG with a median of 50.0 (50.0, 57.5)% of MA according to control.

**Figure A2:**
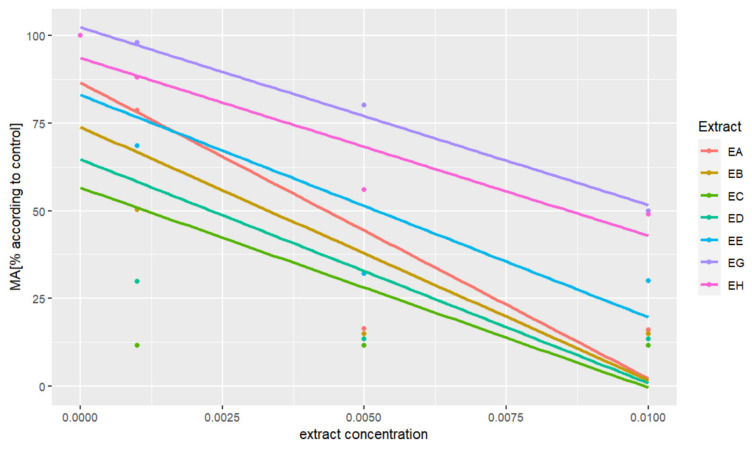
Correlation of extract concentration and cancer cell metabolic activity in % according to control of different hemp extracts.

The metabolic activity of all tested cells decreased in the concentration range from 0 to 0.01 mg/mL, but at higher concentrations of the extract the metabolic activity stabilised in the median range corresponding to the control, which is also shown on Figure A2. The Spearman correlation test showed a statistically significant, strong, inverse relationship between extract concentration and metabolic activity of cells in the range of 0 to 0.01 mg/mL (r = −0.767, *p* < 0.001).

**Figure 5:**
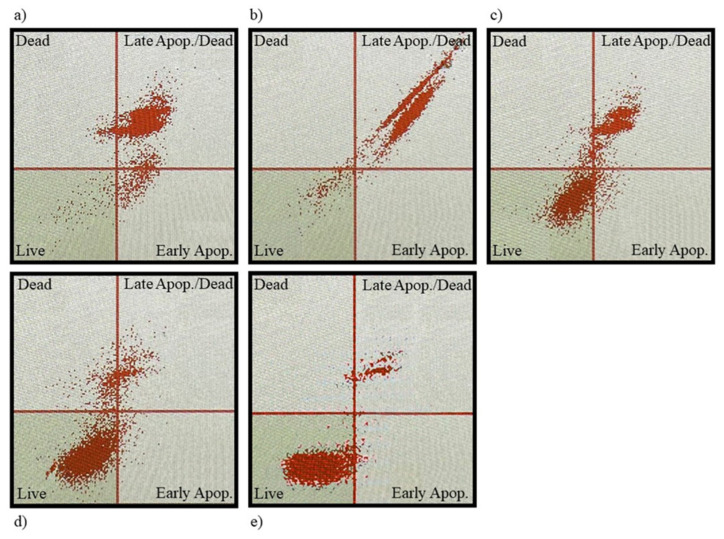
Annexin V/7-AAD staining in melanoma WM-266-4 cells. Cells treated with EC extract of concentration (**a**) 3 × 10^−3^ mg/mL, (**b**) 2 × 10^−3^ mg/mL, (**c**) 10^−3^ mg/mL, (**d**) 7 × 10^−4^ mg/mL and (**e**) untreated cells.

To investigate the extent of apoptotic cell death, WM-266-4 cells were treated with EC extract at different concentrations (*c*_1_ = 3 × 10^−3^ mg/mL, *c*_2_ = 2 × 10^−3^ mg/mL, *c*_3_ = 10^−3^ mg/mL, *c*_4_ = 7 × 10^−4^ mg/mL) and cells were stained with Muse™ Annexin V & Dead Cell Reagent and recorded using the Muse™ Cell Analyzer. Representative results of the assay with untreated WM-266-4 cells are represented in Figure 5e) and WM-266-4 cells treated with EC extract of following concentration are shown: *c*_1_ (a), *c*_2_ (b), *c*_3_ (c) and *c*_4_ (d).

**Figure 6:**
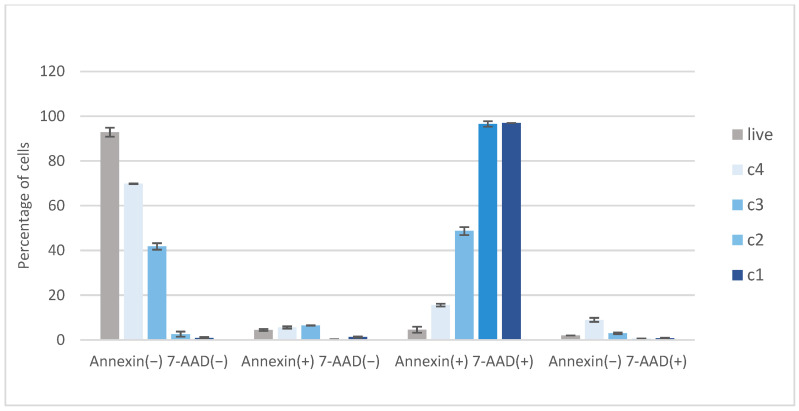
Cell distribution; effect of hemp (EC) extract on activation of apoptosis in WM−266−4 cells (*c*_1_ = 3 × 10^−3^ mg/mL, *c*_2_ = 2 × 10^−3^ mg/mL, *c*_3_ = 10^−3^ mg/mL, *c*_4_ = 7 × 10^−4^ mg/mL).

The graph in Figure 6 shows the percentage of live, early apoptotic, late apoptotic and cellular debris represented by Annexin(−)7-AAD(−), Annexin(+)7-AAD(−), Annexin(+)7-AAD(+) and Annexin(−)7-AAD(+), respectively. Data are presented as mean ± SD of three independent experiments.

**Figure 7:**
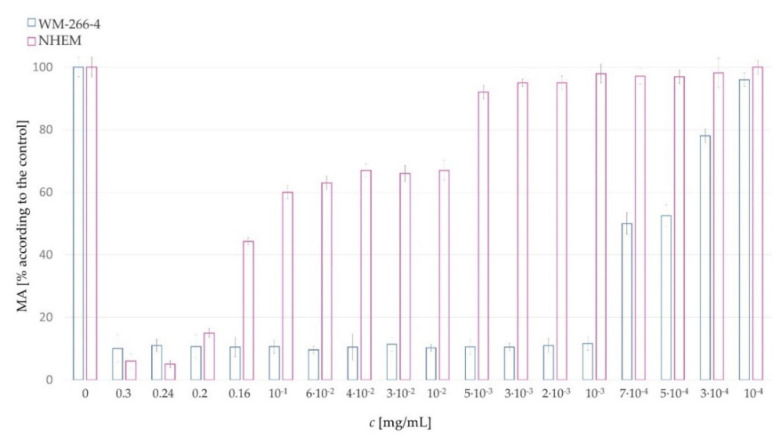
Metabolic activity of melanoma cells WM−266−4 at different concentrations of EC.

The most potent hemp extract (EC) was used for measurements at a higher concentration (Figure 7). The EC extract was applied to melanoma cells WM-266-4 and compared with normal human epidermal melanocytes (NHEMs). NHEMs are pigment-producing cells located at the basal level of the epidermis. Their function is to communicate with keratinocytes via cellular processes (dendrites). Melanocytes produce melanin (a pigment), which is transferred to the keratinocytes and stored in melanosomes located around the nucleus for protection against UV radiation [38].

**Figure 8:**
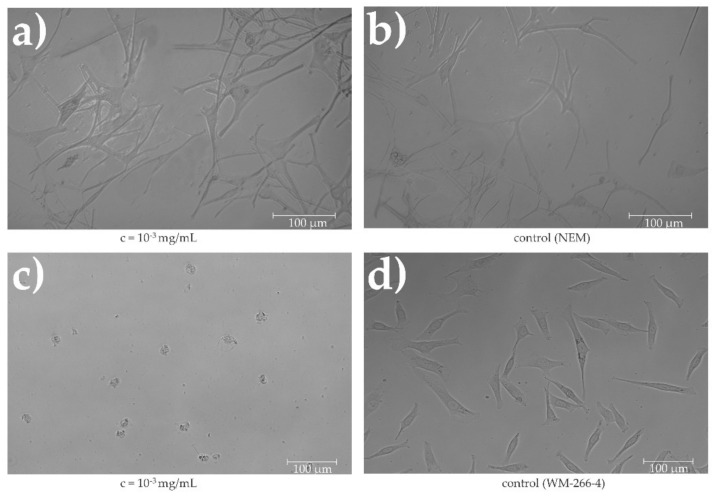
(**a**) Normal human epidermal melanocytes in medium; (**b**) normal human epidermal melanocytes with applied extract (10^3^ mg/mL); (**c**) human melanoma cells WM-266-4; (**d**) human melanoma cells WM-266-4 with applied extract (10^−3^ mg/mL).

In Figure 7, it can be seen that the metabolic activity of cancer cells (WM-266-4) at an extract concentration of 10^−3^ mg/mL is only approximately MA = 11.60%, whereas normal human epidermal melanocytes had a metabolic activity of 97.05% at this concentration. There was no significant difference with the control (MA = 100%). An example of cells morphology (WM-266-4 and NHEMs) using EC extract at 10^−3^ mg/mL is shown in Figure 8. Figure 8b,d shows the controls, i.e., the cells in the complete medium. The morphology of NHEMs when the extract is applied at a concentration of 10^−3^ mg/mL (Figure 8a) and that of the control samples are the same. In contrast, there is a significant difference in the morphology of metastatic cells. When the extract was applied to metastatic cells (Figure 8c), they lost their original morphology.

EC extract was confirmed as the best for our work in these studies. The selected screen fraction (material C) of dried industrial hemp was extracted using two methods (by supercritical fluid CO_2_ at the 350 bar and 60 °C and by ultrasonic extraction with EtOH); both extracts were mixed in a 1:1 ratio. In the current study, dried hemp was further decarboxylated, which contributed to an even higher cannabinoid content in the extract (e.g., the CBD content was measured at 49.5%).

An anticytotoxic effect was also observed for EC extract. When applied to melanoma cells WM-266-4 and normal human epidermal cells (at a concentration of 0.001 mg/mL), it showed significant inhibition of melanoma (MA = 11.7%) and at the same time no effect on normal cells (MA = 97.1%).

## 4. Conclusions

The study was concerned with the determination of optimal procedures for the production and extraction of industrial hemp material. The results show that hemp screening plays a crucial role in obtaining a high-quality extract. Zero waste is achieved with the prescreening process, which is in line with sustainable development. The study confirmed significant differences between extracts of different plant materials against cancer cells (H(6) = 45.264, *p* < 0.001). For all extracts in the range of 0 to 0.01 mg/mL of the applied extract, a statistically significant, strong, inverse relationship between the extract concentration and the metabolic activity of cells (r = −0.767, *p* < 0.001) was confirmed. The most pronounced anticancer effect was determined for hemp extract (EC). When applied to WM-266-4 cancer cells (*c* = 10^−3^), the EC extract inhibited their activity by 88.3%, which means that there is a possibility that the cells were destroyed. This was also confirmed by the apoptosis results, which showed 97% late apoptosis represented by (+)7-AAD(+) when the extract was applied at a concentration of 10^−3^ mg/mL. However, these results are supported by the results obtained in healthy cells (NHEMs), in which the selected extract did not inhibit their activity. In addition to all these results, the screening process showed the possibility of using the entire plant and reducing waste during processing. The process used is novel in hemp processing. It is assumed that the significant inhibition was achieved precisely because of the high content of the CBD, which was measured at 49.5% in the extract mixture. The content of other cannabinoid compounds was much lower (CBC = 1.40%, CBN = 0.15%, THC = 4.47%), but they have many therapeutic effects on the body, such as anticancer and antimicrobial effects [21,23,39,40].

The process is considered as waste-free and consequently economical, as the sieved fractions yield cannabinoid-rich material. Therefore, less solvent is used. Furthermore, the article provides new solutions for the valorisation of the whole plant, waste and by-products, thus contributing to minimal waste generation or fulfilling the popular “zero waste concept” to meet today’s needs and demands of consumers and society.

## Figures and Tables

**Table 1:** MRM parameters of the LC-MS/MS method.

Analyte	Precursor	Fragment	CE	Fragmentation
CBGA	361	343, 317	10, 10	100
CBDA	359	341, 218.8	10, 30	100
CBD	315.2	193.1, 123.1	20, 36	45
THCA	357.4	313.1, 245.1	10, 20	100
THC	315.2	193.1, 123.1	10, 15	50
CBN	311.2	293.1, 223.1	16, 20	50
CBC	315.2	259.1, 81.1	12, 15	45

**Table 2:** Percentages of fractions obtained by seeding.

Materials of Hemp	A	B	C	D	E	G	H
Percentage of sieved material [%]	100	66.23	3.74	0.75	61.13	27.00	5.30

**Table 3:** Yields obtained after extraction.

Extraction Yields [%]	Hemp Extract Label
5.75 ± 0.23	EA
5.32 ± 0.11	EB
18.95 ± 1.13	EC
19.01 ± 0.99	ED
3.62 ± 0.76	EE
25.44 ± 1.39	EG
2.98 ± 0.67	EH

**Table 4:** Cannabinoids of hemp extracts.

Hemp Extract	Cannabinoids						
	CBC	CBD	CBDA	CBGA	CBN	THC	THCA
	[%] of Components in Extracts						
EA	0.510 ± 0.007	27.137 ± 0.745	1.075 ± 0.033	0.080 ± 0.003	0.053 ± 0.001	0.923 ± 0.012	0.070 ± 0.002
EB	1.053 ± 0.023	45.363 ± 0.621	2.788 ± 0.054	1.348 ± 0.003	0.101 ± 0.001	1.409 ± 0.029	ND
EC	1.399 ± 0.018	49.514 ± 0.922	3.627 ± 0.046	1.071 ± 0.012	0.149 ± 0.007	1.474 ± 0.01	ND
ED	0.912 ± 0.013	39.438 ± 1.508	3.430 ± 0.052	1.436 ± 0.019	0.139 ± 0.003	1.452 ± 0.053	ND
EE	0.736 ± 0.009	36.759 ± 0.483	0.704 ± 0.022	0.027 ± 0.001	ND	0.939 ± 0.016	ND
EG	ND	ND	ND	ND	ND	NDN	ND
EH	0.151 ±0.005	4.437 ± 0.102	3.337 ± 0.086	0.314 ± 0.003	0.301 ± 0.002	0.201 ± 0.016	ND

## Data Availability

Not applicable.
